# A new species of *Quexua* from southeastern Peru (Hymenoptera, Crabronidae)

**DOI:** 10.3897/zookeys.141.1965

**Published:** 2011-10-28

**Authors:** Daniel J. Bennett

**Affiliations:** 1 Division of Entomology, Natural History Museum, and Department of Ecology & Evolutionary Biology, University of Kansas, USA

**Keywords:** Apoidea, apoid wasp, Crabroninae, Crabronini, new species, taxonomy

## Abstract

A distinctive new species of the crabronine wasp genus *Quexua* Pate is described and figured from a single male collected from lowland Amazonian rain forest in southeastern Peru. *Quexua cicra***sp. n.** is the only species in the genus known with a sessile metasoma.

## Introduction

**Figures 1–4.**
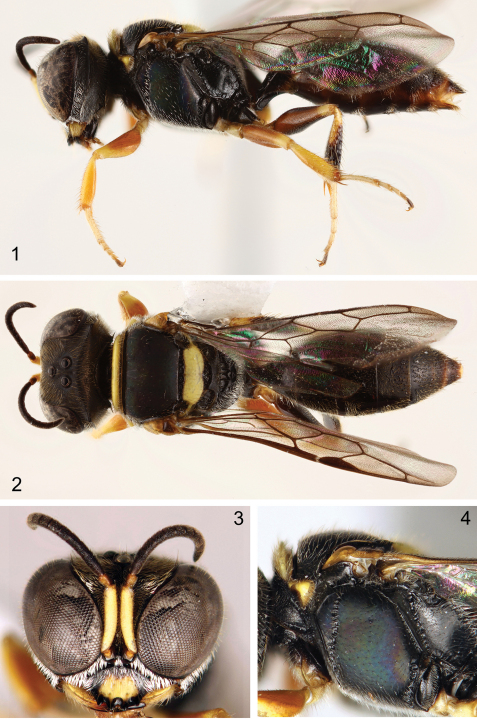
Male holotype of *Quexua cicra* Bennett, sp. n. **1** Lateral habitus **2** Dorsal habitus **3** Facial aspect **4** Mesosoma in lateral view.

The Latin American crabronine genus *Quexua* consists of relatively small, dark wasps found from Costa Rica to the State of São Paulo in Brazil (Leclercq 2002). It is easily recognized by the strong carina spanning the height of the gena which ends dorsally in a distinctive tubercle (Fig. 1). First recognized by Pate (1942), the genus has since been revised several times by (Leclercq (1955, 1980, 2002, 2005). Information concerning its biology is sparse. Cooper (1986) observed a female of *Quexua verticalis* (F. Smith) enter a burrow in a bank which contained a hymenopteran larva and several adult cicadellid prey. According to him, this implied progressive provisioning which, if true, would be unusual for the tribe. Leclercq (2005) indicated that a specimen of *Quexua increta* Leclercq was accompanied by its prey. Apparently disfigured, he supposed it to be a microlepidopteran. Clearly, more observations are needed before much definitive can be said of predatory behavior in the genus. Given the flat form of the pygidial plate throughout the genus, all are likely subterranean nesting; Pate’s (1942) contention that the genus likely includes stem and twig-nesting species is doubtful.

The following provides a description and figures for a new species of *Quexua*recently captured in southeastern Peru. It represents the 17^th^ species of the genus and the ninth known from Peru (Pulawski 2011, Rasmussen and Asenjo 2009). It is unique among the genus in several respects, most notably for its sessile metasoma.

## Material and methods

The single specimen known is deposited in the Museo Nacional de Historia Natural, Lima, Peru (MUSM). Terminology follows Prentice (1998) for morphological structures and Harris (1979) for integumental sculpture. Measurements were made with an ocular micrometer on a Nikon SMZ800 microscope. Images were captured with a Canon 5DII digital camera attached to a Canon EF-S 60mm f/2.8 macro lens. Comparative material was available for the following species: *Quexua cashibo* Pate, *Quexua nericata* Leclercq, *Quexua pano* Pate, *Quexua ricata* Leclercq, and *Quexua verticalis* (F. Smith).

## Systematics

**Genus *Quexua* Pate, 1942**

### Quexua cicra

Bennett sp. n.

urn:lsid:zoobank.org:act:472F527A-D406-4DC7-B146-CBD03FEF3C3A

http://species-id.net/wiki/Quexua_cicra

Figs 1–4 

#### Holotype.

♂, Peru: Madre de Dios: CICRA Field Station, 12.5526°S, 70.1101°W, 295 m, 11–13.VII.2010, Chaboo team, ex. flight intercept trap (MUSM).

#### Diagnosis.

 The sessile metasoma alone will serve to distinguish this species from its congeners (Figs 1–2). Also of diagnostic significance are the relatively long pubescence of the compound eyes (Fig. 3); mat sculpture and blue-green iridescence of the head, metanotum, and mesopleuron (Fig. 4); and lack of a carina along the inner orbit.

#### Description.

*Male:*Body length 7.3 mm (front of compound eye to apex of pygidial plate); head height 1.5 mm, width 1.8 mm; forewing length 4.8 mm (not including tegula); intertegular distance 2.0 mm (including tegulae). Antennal scape elongate, with a single, weak longitudinal carina on inner side of outer surface; flagellum 11-segmented, each article about as long as wide. Clypeus apically with four stout, rounded teeth of similar size (Fig. 3). Toruli separated by less than 1/2 width of torulus, positioned against epistomal sulcus, scapal basin shallow and ecarinate. Facial fovea inconspicuous, roughly size of mid ocellus. Ocellar triangle about as broad as high; mid ocellus slightly smaller than hind ocellus. Gena laterally with strong dorsoventral carina dorsally producing a rounded point. Vertex with narrow pit dorsomedially and low, rounded transverse swelling continuous with more prominent lateral carina of gena. Occipital carina present dorsally, laterally and ventrally; laterally expanded into a lamella and foveate along anterior side (not foveate dorsally), ventrolaterally pointed at a junction between a segment produced towards mandible (mandibular branch of occipital carina of Bohart and Menke (1976)) and a transverse segment meeting the hypostomal margin lateral of midline. Compound eyes converging medially, reaching margins of toruli; with facets larger medially (Fig. 3). Mandible simple and apically acuminate, ventral margin entire (only external surface visible). Labial palpus 4-segmented, first segment longest (about as long as palpomeres II+III); maxillary palpus 6-segmented. Stipes simple, prementum with a median ridge. Pronotal collar rounded, ecarinate, medially slightly depressed (Fig. 2). Mesoscutum with admedian lines distinct but not impressed or ending in foveae, diverging posteriorly; notauli inconspicuous, indicated anteriorly only; parapsidial lines present (Fig. 2). Mesoscutellum and metanotum simple, prescutellar sulcus foveate (9 scallops) (Fig. 2), axillae with sharp edge on inner side. Mesopleuron with postspiracular carina, acetabular carina and omaulus proper present (dorsomedial segment of omaulus absent [that portion mediad junction of omaulus and spiracular carina, crossing omaular area beneath spiracular lobe]); verticaulus, sternaulus, hypersternaulus and mesopleuralus absent; mesepisternal sulcus foveate; scrobe and signum distinct; mesepisternal sulcus foveate over lower 3/4 (Fig. 4). Propodeum anteromedially with six, roughly rectangular enclosures, central pair longest and posterolaterally pointed (Fig. 2); subanteromedially with central fovea surrounded by polished, flat semi-circular disk; anterolaterally with about four longitudinal carinae; lateral propodeal carina well developed. Metapleuron smooth (Fig. 4). Forewing marginal cell squarely truncate apically, about as long as submarginal cell, R1 not much extending beyond apex; 1m-cu (recurrent vein) meeting posterior margin of submarginal cell near latter’s midpoint; cu-a positioned proximal of M + Cu junction by a distance about equal to that of cu-a (Figs 1–2). Hind wing with two closed cells, medial and submedial, latter not quite half as long as former, medial extending to just short of first hamulus; anterior margin with six hamuli, with a distinct elongate, seta (about twice height of basal hamulus) just basal to first hamulus; claval and jugal lobes well-developed, latter rounded apically and reaching nearly to cu-a. Legs simple and fairly slender, pretarsal claws simple, arolia present; fore and mid femora moderately expanded medially, hind femur less so; midtibial calcar distinct, extended beyond apical margin of mid tibia by about half its length. Metasoma simple, sessile, relatively compact, tergum I about 1.1 × longer than posterior breadth (Figs 1–2); tergal graduli apparently absent; tergum I with oblique lateral carina present, laterotergite pendant but not reaching midline of sternum I; pygidial plate more or less quadrate, flat, apex slightly rounded, lateral margin carinate; sternum I with single median ridge over anterior 3/4; sternum VII evenly and fairly broadly rounded apically.

Black except following areas light brown: tegula, proximal 4/5 of fore and mid femora, inner surface of fore tibia, apical tarsomeres, and hind trochanter in part; darker brown are pedicel ventrally, palpi, wing veins, outer surface of hind femur over proximal 4/5 and metasoma laterally and ventrally; yellow areas are entire scape, clypeus largely (broadly over central area, margins black), mandible in proximal half, pronotum, pronotal lobe, mesoscutellum in full, fore coxa anteroventrally, mid coxa lateroventrally, hind coxa narrowly apicoventrally, trochanters, femora apically narrowly (extended basally on posterior surface of fore and meso femora), outer surfaces of fore and mid tibiae, basal 1/3 of outer side of hind femur, and preapical tarsomeres (Figs 1–3). Wings iridescent and mostly hyaline, browned along leading marginal areas of forewing apically. Mesopleuron (Fig. 4), metanotum and (to a lesser a degree) gena and vertex with a distinct blue-green iridescence.

Pubescence overall sparse, mostly off-white to silver, light brown on metasoma and dorsal portions of head and mesosoma; clypeus laterally with fairly dense silver setae, medially mostly bare except for about four prominent, fairly long, thin, brown setae; compound eyes with sparse yet distinctive, long setae (length equal to about half ocellus diameter) (Fig. 3); propodeum with moderately dense silver setae posterolaterally; wings moderately densely setose apically, less so basally; legs sparsely to moderately spinose, the latter particularly on hind tibia. Metasomal tergum I sparsely setose, remaining terga moderately setose, becoming more dense posteriorly; sterna II–VII sparsely setose posteriorly, with a few conspicuously long setae sublaterally.

Surface sculpture overall very fine. Gena, vertex, mesonotum, mesopleuron, mesoscutellum and metanotum dull mat, weakly and sparsely punctate (Fig. 2). Propodeum, metapleuron, legs, sterna (more or less) and terga nitid (characterization of the terga possibly in error, at least partially influenced by residue); tergum I smooth, sparsely and weakly punctate; sterna II–VII imbricate.

*Female*: Unknown.

#### Etymology.

The specific epithet refers to the field station adjacent to the type locality of this species and is treated as a noun in apposition. CICRA (also known as Los Amigos Biological Station) is an acronym for Centro de Investigación Capacitación Río Los Amigos.

#### Comments.

This is the first species of the genus known to have a sessile metasoma. In all others, the length of the first metasomal tergum is distinctly greater than its posterior breadth. Also notable, and possibly unique, are the widespread mat, iridescent sculpture and the length of pubescence of the compound eyes (at least much greater than the *Quexua*species listed above). It is hoped that a female is soon captured and that biological observations of the genus at large will be made.

## Supplementary Material

XML Treatment for Quexua cicra
